# 

**Published:** 1886-03-20

**Authors:** 


### TABLE OF CONTENTS.

ORIGINAL AND SELECTED ARTICLES.

Report of a Case—Death in a Few Hours—What Was It, by J. A. Andrey, M. D., Pineville, North Carolina ... 81
Commencement Exercises of the Southern Medical College—Session of 1885-86 ... 84
The Treatment of Hemorrhoids, F. H. Rorick, M.D., Wauseon, Ohio ... 90
Artificial Dilatation of the Uterine Cervix During Labor, by Dr. W. R. Chittick, Detroit, Michigan ... 93
Bryonia, by Dr. C. S. Lombard, Michigan ... 95
A Case of Placenta Prævia Treated by the Tampon and Version, by Dr. H. C. Archibald, Philadelphia ... 96
Heredity ... 97

ABSTRACTS AND GLEANINGS.

Hyoscine Hydrobromate ... 98
The Galvano-Cautery as an Effectual Treatment of Diphtheria ... 99
Fractures ... 100
The Thermo-Cautery with Treatment of Spontaneous Gangrene; Passage of a Large Piece of Glass Through the Foot After Five Months ... 101
Hypodermic Injections of Cold Water in Sciatica; Hypnone—A New Hypnotic ... 102
Keloid ... 103
Abortion from Sassafras; Removal of Blade of Tooth-Forceps from the Right Bronchus ... 104
Opium Habit; Antipyrine in Epistaxis ... 105
Small-Pox; Peppermint vs. Cocaine; Conium as a Nerve Stimulus ... 106
Amblyopia Nicotiania; The Best Vehicle for Quinine Administered to Children; Ice to the Spine in Obstinate Vomiting; Determination of the sex of the Fœtus ... 107
Abortive Treatment of Gonorrhœa; Treatment of Ottorrhœa; A New Electric Plant; Morphine in Infantile Eclampsia ... 108
Hot Cloths for Uterine Hemorrhage; Glycerin in Colds; Restoring Gray Hair to its Original Color; For Hay-Fever and Coryza; Coryza; The Action of Quinine on the Fœtus ... 109
External Use of Lobelia Inflata; Exenteration of the Eye; Borated Vaseline in Erysipelas; Diphtheria ... 110

SCIENTIFIC ITEMS.

Rattlesnakes; Wonders of the Sea ... 111
The Cobra Poison; Cleaning Hands After Laboratory Work; An Egg Register ... 112

PRACTICAL NOTES AND FORMULÆ.

Tonic and Alterative; Turpine; Mucilage; Pimple Ointment; Chilblain Remedy ... 113
Hypodermic Quinine Solution; Carson's Paint; Iodine in the Treatment of Chronic Rheumatism; Superior Tonic Mixture; Syrup of White Pine ... 114

EDITORIALS AND MISCELLANEOUS.

Death of Dr Hamilton; Death of Jules Guerin; Dr. Asbel Smith; Medical Association of Ga. 115
American Medical Association; Medical Society of Tennessee; Association of American Editors; The Country Doctor ... 116
Death of Dr. Caldwell, of Atlanta ... 117
Dr. Andrey's Case; Books and Pamphlets Received ... 118
Receipted; Special Notices ... 120

